# Easy-to-Use Visual Sensing System for Milk Freshness, Sensitized with Acidity-Responsive N-Doped Carbon Quantum Dots

**DOI:** 10.3390/foods11131855

**Published:** 2022-06-23

**Authors:** Xuetao Hu, Xinai Zhang, Yanxiao Li, Jiyong Shi, Xiaowei Huang, Zhihua Li, Junjun Zhang, Wenting Li, Yiwei Xu, Xiaobo Zou

**Affiliations:** 1 School of Food and Biological Engineering, Jiangsu University, Zhenjiang 212013, China; xuetaojsu@ujs.edu.cn (X.H.); zhangxinai@mail.ujs.edu.cn (X.Z.); huangxiaowei@ujs.edu.cn (X.H.); lizh@ujs.edu.cn (Z.L.); 2111918019@stmail.ujs.edu.cn (J.Z.); 2112018022@stmail.ujs.edu.cn (W.L.); zou_xiaobo@ujs.edu.cn (X.Z.); 2 Analyzing and Test Center, Jiangsu University, Zhenjiang 212013, China; 1000003438@ujs.edu.cn; 3 School of Food Science and Technology, Henan University of Science and Technology, Zhengzhou 450001, China; xuyiwei@ujs.edu.cn

**Keywords:** milk freshness, acidity, fluorescent sensor, carbon quantum dots, food safety

## Abstract

This study established a flexible and eye-readable sensing system for the easy-to-use, visual detection of milk freshness, using acidity-responsive N-doped carbon quantum dots (N-CQDs). N-CQDs, rich in amino groups and with characteristic acidity sensitivity, exhibited high relative quantum yields of 25.2% and an optimal emission wavelength of 567 nm. The N-CQDs fluorescence quenching upon the dissociated hydrogen ions (H^+^) in milk and their reacting with the amino groups produced an excellent linear relation (R^2^ = 0.996) between the fluorescence intensity and the milk acidity, which indicated that the fluorescence of the N-CQDs was highly correlated with milk freshness. Furthermore, a fluorescence sensor was designed by depositing the N-CQDs on filter-papers and starch-gel films, to provide eye-readable signals under UV light. A fluorescence colorimetric card was developed, based on the decrease in fluorescence brightness as freshness deteriorated. With the advantages of high sensitivity and eye readability, the proposed sensor could detect spoiled milk in advance and without any preprocessing steps, offering a promising method of assessing food safety.

## 1. Introduction

Fresh milk is rich in nutrients and plays an important role in daily life [1]. However, it is particularly susceptible to spoilage during its production, transportation, processing, and marketing because it provides a favorable growth substrate for various decay-inducing microorganisms (e.g., pathogens and spoilage organisms, etc.) [2,3]. The denaturation of proteins, rancidity of fats, hydrolysis of carbohydrates, and other spoilage reactions that are caused by microbial fermentation, cause flavors to disappear and nutritional value to decrease, resulting in huge food waste and financial losses. Moreover, the formation of harmful substances and the reproduction of microbial pathogens can lead to food poisoning [4]. Thus, it is of great practical significance to evaluate milk freshness to ensure milk safety and human health. The microorganisms present in milk begin to break down lactose into lactic acid, leading to an increase in acidity during storage [5]. Milk with an acidity > 18 °T is considered to be spoiled and unfit for consumption, indicating that microbes are growing and multiplying in large numbers [6]. Therefore, acidity can be used as the most important freshness indicator in evaluating milk freshness.

Traditional methods for the detection of milk acidity include neutralization titrations (phenolphthalein indicator titration) [7] and potentiometers (pH meter) [8,9]. These methods have been successfully applied in the accurate detection of milk freshness [7,9]. However, electrode-based methods and acid-based titration techniques are not suitable for low volume detection and on-site monitoring. Certain rapid methods, such as near-infrared spectroscopy and spectrophotometry, still involve tedious operations and poor repeatability. Recently, fluorescence sensing methods have been widely used for food assessment, owing to their high operational simplicity, high sensitivity, and short response time [10,11]; however, these fluorescence methods need to be improved, owing to the requirement of specialized instruments [12]. Eye-readable sensors have attracted increasing attention for their simple and convenient assessment, without the need for electronics and external digital readouts [13,14]. Such sensors have been fabricated using organic fluorophores, such as fluorescein isothiocyanate (FITC) [8] and succinimide substituted 2-(2′-hydroxyphenyl) benzothiazole derivative (BTSA) [15]. These fluorescent dyes, which have good fluorescence emission performance, often suffer from certain disadvantages, including poor biocompatibility, broad red-tailed photoluminescence spectra, and high susceptibility to photobleaching [16], prompting us to improve the fluorescence properties of eye-readable sensors.

**Figure 1:**
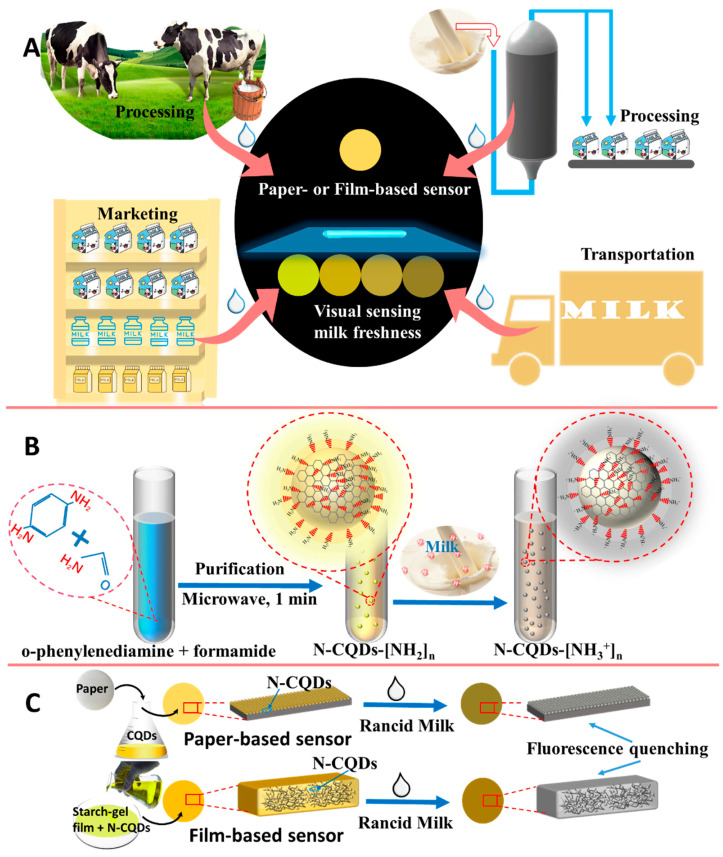
Schematic illustration of (**A**) easy-to-use visual sensing of milk freshness, using fluorescence sensors based on N-CQDs; (**B**) preparation of carbon quantum dots (N-CQDs); and (**C**) the fluorescence quenching of sensors by rancid milk.

Carbon quantum dots (CQDs) are a new type of fluorescent material that have been applied for food assessment [17], biological analysis [18], and environmental monitoring, [19] owing to their excellent optical properties and biocompatibility [20]. Previous studies have shown that CQDs exhibit excellent responses to pH variations and can be used for the detection of pH and organophosphorus pesticides [17,21,22]. However, there have been relatively few scientific studies regarding freshness associated with acidity sensing using CQDs. Therefore, this study aimed to develop flexible and eye-readable fluorescence sensors to monitor milk freshness using acidity-responsive N-doped CQDs, thus, simplifying the experimental equipment and procedures required for the fluorescence monitoring of milk freshness during processing, transportation, and marketing (Figure 1). N-CQDs were synthesized from poly-amino compounds, resulting in rapid responses to rancid milk. The fluorescence of the fabricated sensors can be quenched by the rancid milk and, thus, can be used for milk freshness assessments. These low-cost and easy-to-use fluorescence sensors, which are portable and eye-readable, can be used for the visual sensing of milk freshness, with the aid of only UV light and the naked eye.

## 2. Experimental Section

### 2.1. Materials and Apparatus

Chemical reagents (O-phenylenediamine, formamide, quinine sulfate (QS), and sodium hydroxide, etc.) and materials (filter paper and cow’s milk, etc.) in this study have been described in the experimental section of the Supplementary Materials file. The employed apparatus has also been introduced in the Supplementary Materials file.

### 2.2. Preparation of Acidity-Responsive N-CQDs

This preparation was performed according to previous studies, with minor modifications [23,24]. O-phenylenediamine (0.5 g) was dissolved in 10 mL of formamide, and the mixed solution was heated in a microwave oven (750 W, Midea, Tianjin, China) for 1 min to obtain a dark yellow solution. Large impurities in the solution were removed using a 0.22 μm filter paper. The unreacted o-phenylenediamine and formamide were removed by silica gel column chromatography [25]. Silica gel powder dissolved in dichloromethane (*v*/*v* = 1:2) was used as the adsorbent. After the unpurified N-CQDs solution was completely absorbed, a mixture of ethanol and ethyl acetate (volume ratio: 1:1) was added as an eluent to collect the pure N-CQDs solution. The effluent flow obtained in the first and last three minutes was discarded. Pure N-CQDs powder, obtained by vacuum freeze-drying, was re-dissolved in 10 mL of purified water to obtain the original N-CQDs solution.

### 2.3. Investigation of Fluorescence Change Mechanism

Acid buffer solutions with different levels of pH (4.5, 5, 5.5, 6, 6.2, 6.4, 6.6, 6.8, 7.0, and 7.2) were prepared, and 1.8 mL of buffer solution was mixed with 0.2 mL of the original N-CQDs solution. After standing for 1 min, the mixture was poured into a quartz colorimetric cell. UV-vis absorption and fluorescence spectra of the N-CQDs in different buffer solutions were recorded. The mechanism of the fluorescence change in the N-CQDs was revealed by the fluorescence and absorption properties.

### 2.4. Establishment of Fluorescence Method

Lactic acid solutions with different acidities (12.1, 14.7, 17.6, 21.0, 24.5, 28.8, 31.8, 34.6, and 40.8 °T) were prepared. Each lactic acid solution (0.2 mL) was mixed with the N-CQDs solution (1.8 mL) and left standing for 3 min. The fluorescence spectra of the mixtures with different levels of acidity were recorded and the relation between acidity and fluorescence intensity was investigated by linear regression. Milk samples at different acidity levels (11.6, 13.7, 17.9, 21.5, 24.5, 27.8, 30, 34.2, and 40.2 °T) were prepared by adding lactic acid. Next, the 200 μL of N-CQDs solution was mixed with the milk sample (1.8 mL). Fluorescence spectra of mixtures poured into a quartz colorimetric dish were recorded after standing for 3 min. A linear equation was established on the basis of the relation between milk acidity and fluorescence intensity.

### 2.5. Fabrication of Visual Fluorescence Method

To prepare the paper-based sensor, filter papers (circles, 2 cm in diameter) were immersed in the N-CQDs solution, at the optimal concentration, for 2 min. To prepare the film-based sensor, 2 g of starch, 2 g of polyvinyl alcohol, and 1 mL of glycerol were added to 100 mL of distilled water and heated for 35 min at 90 °C. Thereafter, N-CQDs solution was added to the cold film-forming solution at the optimal dosage. Subsequently, 18 mL of film solution was spread on a glass petri dish, 60 mm in diameter. The paper-based and film-based sensors were dried at 40 °C in an oven for 10–12 h. These sensors were vacuum packaged and stored in the dark at room temperature. After preparing the two types of sensors, milk (50 μL) at different acidity levels (11.6, 13.7, 17.9, 21.5, 24.5, 27.8, 30, 34.2, and 40.2 °T) was added to the sensor. Finally, images of the paper-based and film-based sensors indicating milk acidity were combined as acidity colorimetric cards.

### 2.6. Fluorescence Monitoring of Milk Freshness

Fresh, sealed cow’s milk was opened and divided into 24 conical bottles, under aseptic conditions. Next, they were re-sealed and stored at 37 °C for 0, 2, 4, 6, 8, 10, 12, and 24 h. Three samples were collected at each time point. Acidity sensing was performed under optimal reaction conditions. Milk samples (0.2 mL) were mixed with the N-CQDs solution (1.8 mL), stirring evenly. Fluorescence spectra of these mixtures were recorded 3 min later. Milk acidity was calculated using the fluorescence intensity. Milk freshness was evaluated by fluorescent sensors, following the procedures described in the above section.

### 2.7. Standard Methods for Evaluating Milk Freshness

To verify the accuracy of the developed methods, the phenolphthalein indicator titration method and a sensory evaluation were performed to measure the acidity and freshness of milk samples [26]. The acidity of lactic acid solution and milk was measured as follows: milk (10 g) was diluted with 20 mL of freshly boiled water and cooled to room temperature. After the addition of 0.5% phenolphthalein indicator (2 mL), the diluted milk was titrated with 0.1 mol/L of NaOH standard solution. Titration was not stopped until slightly red and the red color was maintained for 30 s. The volume of NaOH consumed was substituted into Equation (S1) (X(°T) = 100 cV/0.1 m) to calculate the acidity of the milk (°T) (supporting information). Nine trained judges were selected to evaluate the freshness of the milk samples. The freshness attributes were evaluated by fresh (good quality), spoiling (approaching spoiled), and spoiled (poor quality). All samples were served once and their order of serving was randomized.

## 3. Results and Discussion

### 3.1. Structural Characterization of N-CQDs

**Figure 2:**
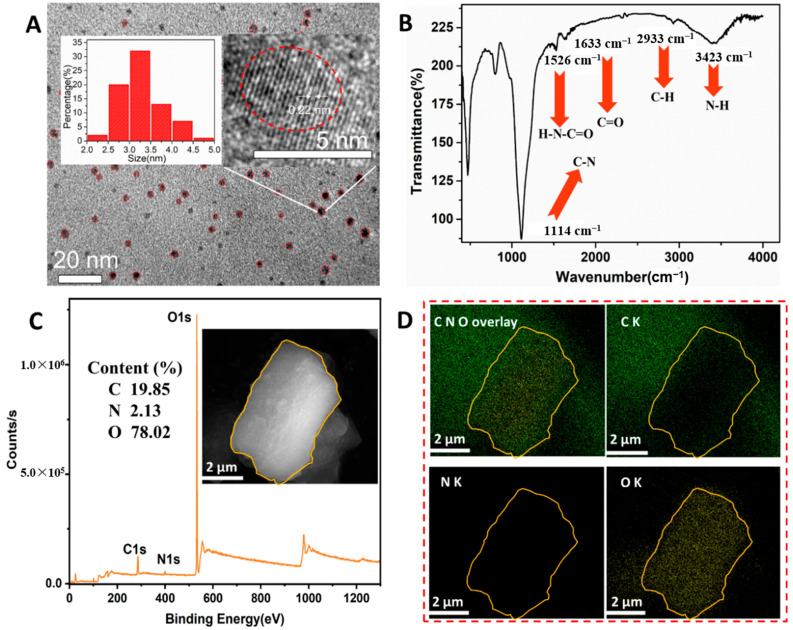
(**A**) TEM image of N-CQDs, inset: diameter distribution of N-CQDs and high-resolution TEM of a single N-CQD particle; (**B**) FT-IR spectrum of N-CQDs; (**C**) XPS spectrum of N-CQDs, inset: scanning electron microscopy (SEM) image of N-CQDs powder; and (**D**) EDS mapping images of elements on the surface of N-CQDs.

The N-CQDs were prepared from o-phenylenediamine and formamide, using the microwave assisting method (Figure 1A). FT-IR spectra, XPS spectra, and EDS mapping images were obtained to investigate their morphology, active groups, and the constituent elements of the N-CQDs (Figure 2). TEM images revealed that the N-CQDs had a regular spherical shape (Figure 2A), with a range of 2.2–4.7 nm in diameter and an average value of 3.2 nm (left inset in Figure 2A), which is indicative of the uniform size of the N-CQDs. The right inset showed a high-resolution TEM image of a single N-CQD particle, which clearly revealed that the N-CQDs had a good crystalline structure, with a lattice fringe spacing of 0.22 nm. The FT-IR spectrum of the N-CQDs showed characteristic peaks, centered at 3423, 2933, 1633, 1526, 1114, and 471 cm^−1^ (Figure 2B). The peaks at 3423, 2933, and 1633 cm^−1^ were attributed to the nitrogen–hydrogen bonds (NH_2_), carbon–hydrogen bonds (C-H), and amide–carbonyl bonds (H2N-C=O), respectively. The peak at 1526 cm^−1^ was due to the stretching vibration of the carbon–oxygen double bonds (C=O) or to the carbon–nitrogen double bonds (C=N). The results indicated that the surface of the N-CQDs was rich in a large number of active groups (e.g., amine, carboxyl, etc.).

The elemental composition and distribution of the N-CQDs were analyzed by XPS and EDS mapping (Figure 2C,D). The inset of Figure 2C shows an SEM image of the N-CQDs powder, used for analysis. The XPS spectrum of the N-CQDs (Figure 2C) displayed that three binding energy peaks, centered at 285.94, 398.99, and 532.49 eV, were assigned to the carbon (C), nitrogen (N), and oxygen (O) elements, with relative contents of 19.85%, 2.13%, and 78.02%, respectively (Table S1). The XPS spectrum of the C1s (Figure S1A) displayed three peaks of 285.0, 286.0, and 288.0 eV, generated from the C-C, C-N and C=O groups, respectively. The N1s spectrum (Figure S1B) presented three peaks of 399.1, 400.5, and 401.3 eV, caused by the nitrogen in pyridine, amino, and pyrrole, respectively. The XPS spectrum of the O1s demonstrated that the characteristic binding energy peaks were centered at 531.7 and 533.0 eV, and their corresponding characteristic groups were C=O and C-OH and C-O-C, respectively (Figure S1C). In addition, EDS mapping was used to analyze the elemental distribution of the N-CQDs (Figure 2D). The first image is a superposition image of the C, N and O elements. The distribution of the C, N and O elements on the N-CQDs surface is displayed in the last three images of Figure 2D. These results further demonstrated the successful synthesis of N-CQDs, which are composed of evenly distributed C, N and O elements, and are rich in amine and carboxyl groups.

### 3.2. Optical Properties of N-CQDs

**Figure 3:**
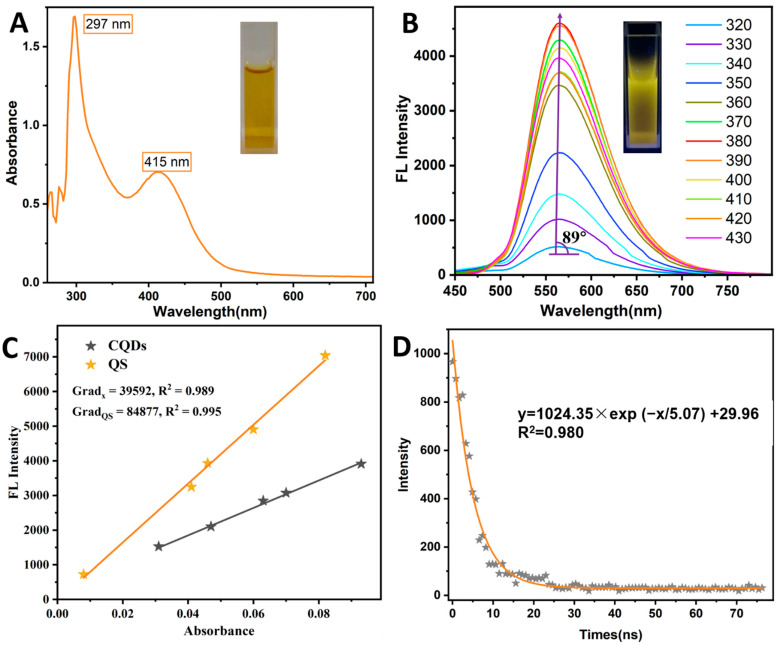
(**A**) UV-vis spectrum of N-CQDs, inset: image of N-CQDs under natural light; (**B**) Fluorescence spectra of N-CQDs, under excitation of different wavelengths, inset: fluorescent image under UV light (365 nm); (**C**) Fluorescence lifetime scatter plot of N-CQDs and its fitted curve; (**D**) Plots of fluorescence intensity vs. absorbance of N-CQDs and QS.

The UV-vis and fluorescence spectra, fluorescence lifetime, and relative fluorescence quantum yields (QYs) were analyzed to verify the excellent optical properties of the N-CQDs, especially their fluorescence (FL) characteristics (Figure 3). As shown in Figure 3A, the N-CQDs exhibited obvious characteristic absorption peaks at 297 and 415 nm, which were attributed to π−π* transitions of sp2-conjugation in the carbon core and n−π* transition of functionalized molecules or doped atoms on the N-CQDs surface, respectively [24]. The N-CQDs solution was observed to be yellow under natural light (inset of Figure 3A). The emission spectra of the N-CQDs, excited at different wavelengths (320–430 nm), showed that the optimal excitation and emission wavelengths were 380 nm and 567 nm, respectively (Figure 3B). The solution displayed distinct yellow fluorescence under a UV light of 365 nm (inset in Figure 3B). The emission wavelengths were excitation-dependent; however, the red shift of the emission wavelength, with an increasing excitation wavelength, was not significant because of the 89°angle between the emission wavelength tendency line and the horizontal line. This may be due to the fact that the N atoms doped on the surface of the N-CQDs inhibited this shift [24].

In this study, formamide acted as a passivator to synthesize the N-CQDs, as such, the increase in the N content was able to change the defect in the energy band to improve the FL efficiency [18]. Both the relative QYs and the fluorescence lifetime (τ) were measured to evaluate the FL efficiency of the N-CQDs. τ is the time required for the FL intensity to drop to 1/e of the maximum FL intensity in the excited state [27]. Figure 3C shows a fluorescence lifetime scatter plot of the N-CQDs, which was fitted by single, double, and triple exponential function. It was found that the fitting result of the single exponential function was optimal, and the τ of the N-CQDs was 5.07 ns. The fitting curve equation was y = 1024.35exp(−x/5.07) + 29.96 (R^2^ = 0.98). The fluorescence QY was an important index for evaluating FL efficiency. Quinine sulfate (with QYs of 54%) was used as the standard material to measure relative QYs. In Figure 3D, the black fitting curve exhibited the linear relation between the absorbance and the FL intensity (a.u.) of quinine sulfate, while the red fitting curve illustrated the linear relation between the absorbance and the FL intensity (a.u.) of N-CQDs. The QY of the N-CQDs was calculated to be 25.2%, by substituting the slopes of the two fitting curves into Equation (S2) [Q_x_ = Q_st_ (A_st_/A_x_) (I_x_/I_st_) ((η^2^_x_)/(η^2^_st_)], as described in the supporting information. As shown, the FL properties were excellent and better than some of the previously reported N-CQDs, because of the N doping [24]. The N atoms can be protonated, and this proton transfer to the conjugated C structure may result in FL enhancement of the N-CQDs [28].

### 3.3. FL Response to Solutions with Various pH

**Figure 4:**
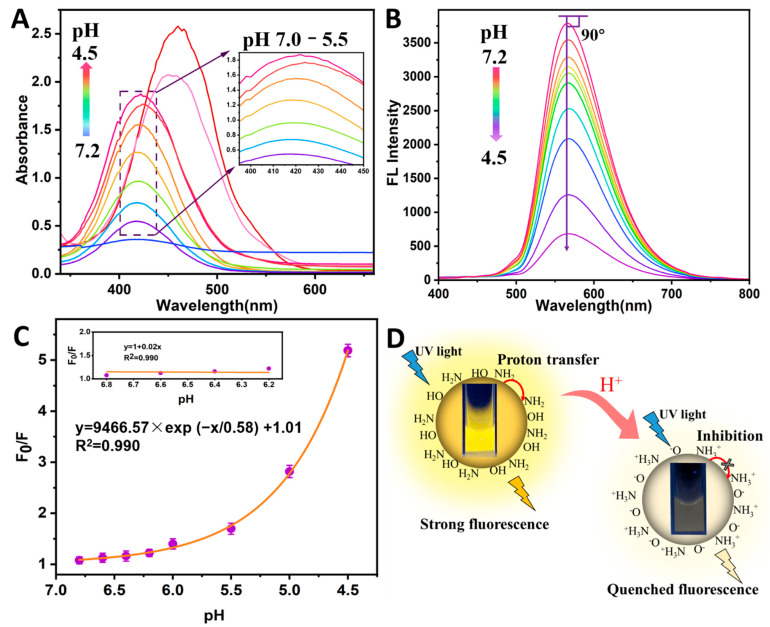
(**A**) UV-vis absorption spectra of N-CQDs solution at different pH values (4.5, 5, 5.5, 6, 6.2, 6.4, 6.6, 6.8, 7.0, and 7.2); (**B**) Fluorescence spectra of N-CQDs solution at different pH values; (**C**) Exponential fitting curve between F_0_/F and pH values (7.0–4.5), inset: linear curve between F_0_/F and pH values (6.8–6.2), the error bars were equal to the standard deviation of three measurements; (**D**) Schematic illustration of fluorescence quenching of N-CQDs.

The UV-vis absorption and fluorescence spectra of the N-CQDs in solutions with different pH values (4.5, 5, 5.5, 6.0, 6.2, 6.4, 6.6, 6.8, 7.0, and 7.2) were explored to demonstrate the acidity-sensitive characteristics, revealing the FL response mechanism (Figure 4). The UV-vis absorption spectra of the N-CQDs under different pH conditions are displayed in Figure 4A. The absorbance at 415 nm was regularly increased, and their peak wavelengths remained unchanged as the pH value changed from 7.0 to 5.5. However, the absorption peak’s red shift was obvious at a pH < 5.5. As shown in Figure 4B, the FL emission wavelength was maintained at approximately 567 nm under different pH values (7.2–4.5). There was no emission wavelength shift, owing to the 90-degree angle between the emission wavelength tendency line and the horizontal line. Moreover, the N-CQDs were sensitive to H^+^, as indicated by the quenching of the FL intensity with decreasing pH values. Fluorescence quenching is typically divided into dynamic and static quenching [29]. If the FL quenching process is treated as dynamic quenching, the interaction relationship between the N-CQDs and the quencher molecules (H^+^) can be described by the Stern–Volmer (SV) equation (Equation (S3): F_0_/F = 1 + K_SV_ [Q] = 1 + k_q_τ_0_ [Q], supporting information) [23,30].

The FL quenching mechanism involving N-CQDs and H+ was analyzed by SV fitting and optical characteristic peaks (Figure 4C,D). The F_0_/F value increased exponentially, as the pH value decreased (F_0_/F = 9466.57exp(−V_pH_/0.58) + 1.01, Figure 4C). pH values in the range of 6.8–6.2 had a good linear relation with the F_0_/F value, via the SV equation fitting (F_0_/F = 1 + 0.02V_pH_, inset in Figure 4C). The SV equation showed that the value of K_SV_ was 0.02, and k_q_ was calculated to be 6.69 × 106 L/mol·s (τ_0_ = 5.07 ns). The k_q_ value between the N-CQDs and H^+^ was much lower than the limiting diffusion rate constant of the fluorophore (2.0 × 10^10^ L/mol·s), and the UV-vis absorption spectra demonstrated that there was no shift in the range of pH 6.8–6.2. These results indicate that the FL quenching mechanism at pH 6.8–6.2 is dynamic. The significant red shift at pH 5–4.5 indicates that the FL quench between the N-CQDs and H^+^ occurs via a static quenching process, which was attributed to the formation of the N-CQDs-H complex. In summary, the FL quenching mechanism gradually changed from dynamic to static, with a decrease in the pH value. The FL of the N-CQDs was quenched as a result of the inhibition of the proton transfer from N atoms to the conjugated structure as the pH decreased (Figure 4D). A study concerning the mechanism of FL quenching demonstrated that N-CQDs had a regular response to solutions of pH 7.0–5.5.

### 3.4. N-CQDs-Based Sensitive FL Assay

**Figure 5:**
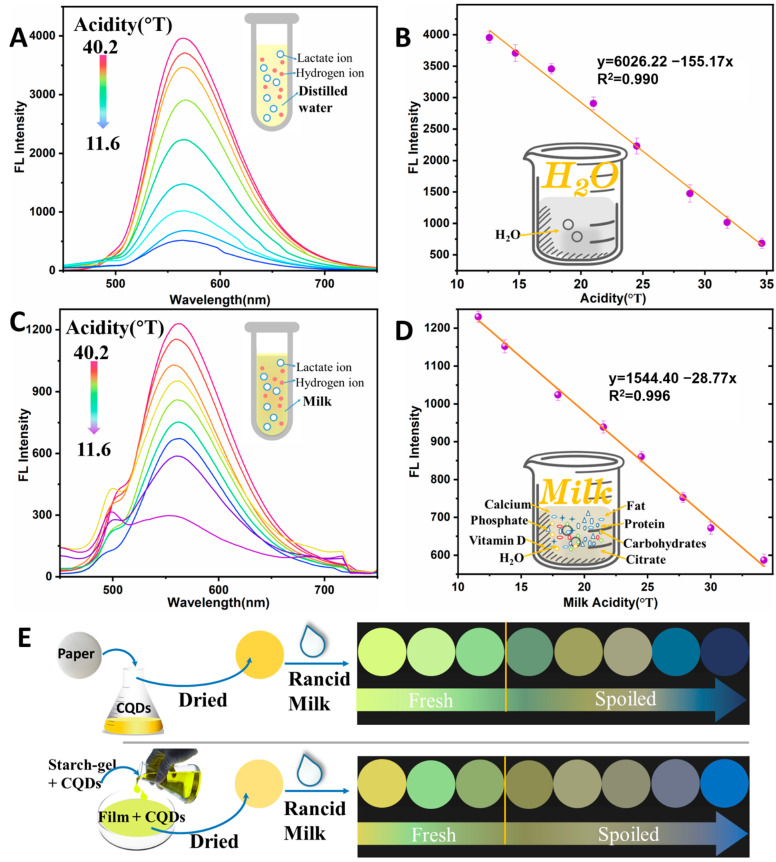
(**A**) Fluorescence spectra of N-CQDs for detecting different levels of acidity, inset: lactic acid–water solution; (**B**) Fitted curve between fluorescence intensity and acidity, the error bars were equal to the standard deviation of three measurements, inset: lactic acid–milk solution; (**C**) Fluorescence spectra of N-CQDs with milk at different levels of acidity, inset: the solvent–water; (**D**) Fitted curve between fluorescence intensity and milk acidity, inset: the solvent–milk; (**E**) Paper- and film-based standard colorimetric card for different milk freshness.

The increase in milk acidity was mainly caused by the production of dissociated hydrogen ions (H^+^) from lactic acid. The relation between the FL intensity and the acidity of the lactic acid–milk and lactic acid–water solutions was investigated for the rapid and sensitive detection of milk acidity. The optimal reaction parameters (N-CQDs dosage and reaction time) are described in the Supplementary Materials (Figure S2). Figure 5A shows the FL spectra of N-CQDs in lactic acid–water solution, under the optimal conditions. The lactic acid–water solution was a mixture of lactic acid, as solute, and water, as solvent (inset in Figure 5A). It can be seen that the maximum FL emission peak of the N-CQDs was 567 nm. The FL intensity at 567 nm decreased, with increasing acidity (12.1, 14.7, 17.6, 21.0, 24.5, 28.8, 31.8, 34.6, and 40.8 °T). A good linear relation was found between the FL intensity at 567 nm and the acidity (12.1–34.6 °T) (Figure 5B). Their linear equation was y = 6026.22 − 155.17x [R^2^ = 0.990, where y is the FL intensity (a.u.), and x is the milk acidity (°T)]. These results indicated that the N-CQDs had a regular response to the acidity of the lactic acid–water solution, without interfering substances (inset in Figure 5B).

The presence of other trace acid substances (e.g., fatty acid, citrate, phosphate, casein, etc.) in milk may interfere with the FL properties of N-CQDs. Milk samples with different levels of lactic acid were investigated to determine the FL response of the N-CQDs (Figure 5C). Figure 5C shows the FL spectra of the N-CQDs in lactic acid–milk solutions at different acidities (11.6, 13.7, 17.9, 21.5, 24.5, 27.8, 30.0, 34.2, and 40.2 °T). The FL intensity at 567 nm gradually decreased, with increasing milk acidity. A new fluorescence peak of approximately 490 nm was attributed to the fluorescent molecules of milk, such as casein, and Vitamins B2 and B6 [31]. By fitting the relation between the FL intensity and the milk acidity, a good linear relation between the two was observed, with a milk acidity range of 11.6–34.2 °T (Figure 5D). Their linear equation was y = 1554.63 − 28.77x (R^2^ = 0.996) where y is the FL intensity, and x is the milk acidity (°T) Compared with the FL characteristics of the N-CQDs with added lactic acid–water solution, the FL intensity decreased by two thirds (from 3982 a.u. to 1237 a.u.), and a new FL peak appeared, due to the interference from protein (especially casein) and fatty acid. The results demonstrated that the milk matrix had a significant influence on the FL characteristics of the N-CQDs. This FL method with an excellent linear coefficient, established by milk as the solvent (inset in Figure 5D). This shows that acidity-sensitive N-CQDs have a regular signal response to different levels of rancid milk, without any negative influence in practical applications. The coefficient of variation of the FL response signal was lower than 3.64%, by measuring the N-CQDs before and after storage, at 4 °C for 30 days. This indicated that the storable N-CQDs could reduce the reagent preparation time and simplify the experimental steps. This FL method was more credible and more reproducible, compared to other detection methods for milk acidity [7,8,13]. As a result, the FL method based on acidity-responsive N-CQDs can be successfully used to detect different levels of acidity in milk, by investigating the FL characteristics of milk samples without any pre-processing steps.

### 3.5. FL-Based Visual Assay

Since there is a high correlation between milk acidity and milk freshness, the acidity-sensitive N-CQDs were further used to fabricate paper- and film-based FL sensors for the rapid and visual sensing of milk freshness. The fabricated sensors were performed using 50 μL of milk samples at different acidity levels (11.6, 13.7, 17.9, 21.5, 24.5, 27.8, 30.0, 34.2, and 40.2 °T). The paper- and film-based standard colorimetric card at different levels of milk freshness were acquired using an FL imaging analyzer, with a UV light of 365 nm (Figure 5E). The FL brightness in paper- and film-based sensors decreased with an increase in milk acidity. As displayed by the paper-based sensor, the bright yellow–green gradually became a light green, then turned a deep yellow, and, finally, turned purple or blue. For the film-based sensors, the deep yellow–green FL changed in the same way as that of the paper-based sensors. However, the FL performance of paper-based sensors was superior to that of film-based sensors because of their wider color range and higher FL brightness. According to the milk safety standard, milk with an acidity >18.0 °T was considered unqualified, and that at >20 °T was badly spoiled and no longer edible [6,32]. The FL brightness characteristics of fresh and spoiled milk are depicted by two color gradient arrows in Figure 5E. The FL brightness from the head to the red vertical line represented fresh milk, and that from the red vertical line to the end indicated that the milk was spoiled. The FL brightness in the presence of different levels of rancid milk was collected, and these color characteristics could rapidly, accurately, and visually indicate milk freshness.

### 3.6. Monitoring of Milk Freshness

**Figure 6:**
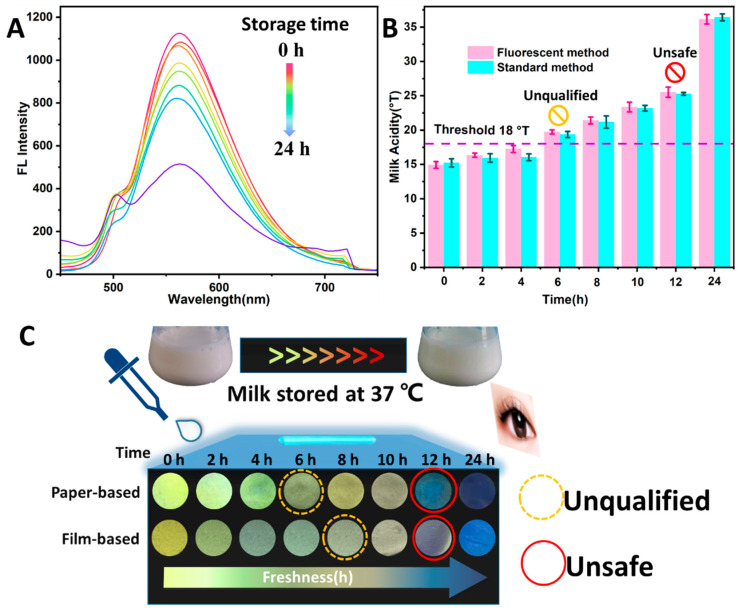
(**A**) Fluorescence spectra of N-CQDs in the presence of milk at different storage times; (**B**) Milk acidity during storage detected by the fluorescence method (pink bars) and standard method (blue bars), Purple dot line represented the milk freshness threshold set in national standard; (**C**) Monitoring milk freshness over different storage times by fluorescence sensors.

Milk, when stored at 37 °C, acted as a practical application sample to verify the feasibility of the developed system. The phenolphthalein indicator titration method and a sensory evaluation were used as the standard means to evaluate the accuracy of the developed system. Figure 6A shows the FL spectra of the N-CQDs added to milk at different storage times (0, 2, 4, 6, 8, 10, 12, and 24 h). It can be seen that the FL intensity at 567 nm gradually decreased as the storage time increased. Milk acidity can be calculated by substituting the FL intensity at 567 nm (F_567_) into the linear equation (acidity = (1554.4 − F_567_)/28.77). The acidity of the milk stored for 0, 2, 4, 6, 8, 10, 12, and 24 h was 14.9, 16.3, 17.2, 19.7, 21.4, 23.36, 25.5, and 36.1 °T, respectively (Figure 6B). The milk acidity at each storage time, measured using the standard method, was 15.2, 15.9, 16.0, 19.3, 21.1, 23.1, 25.2, and 36.4 °T, respectively (Figure 6B). To evaluate the differences between the two methods, a significance analysis (F test) was conducted on data for the two groups, which revealed that the two methods had no significant difference (*p* > 0.05) [33]. According to the freshness threshold set in national standards (purple dot line), milk stored for > 4 h becomes stale. These results showed that milk that had been stored for 6 h was unqualified and that milk that had been stored for 12 h was unsafe. The freshness of milk can be accurately and rapidly monitored using the developed FL method.

On-site sensing is necessary for testing milk freshness during the milk production, processing and sales process. The fabricated FL sensors can be used for the visual sensing of milk freshness without any instrument other than the naked eye and UV light (Figure 6C). The FL brightness of the paper-based sensor changed with the addition of milk samples at different storage times. The milk stored for 4 h was fresh, according to the standard colorimetric card. The acidity of the milk stored for 6 h was >17.9 °T, indicating that the milk was unqualified and approaching spoiled (yellow circle). The acidity of the milk samples stored for 8 h was approximately 21.5 °T, indicating that the milk was spoiled. From the results obtained using the film-based sensors, the acidity of the milk samples after 8 h and 10 h was approximately 17.9 and 21.5 °T, respectively. This revealed that the milk stored for 8 h was approaching spoiled (yellow circle), and that the milk stored for 10 h was spoiled. The paper- and film-based sensors turning blue indicated that the milk acidity for the samples stored for 12 h was >30 °T, indicating that the milk might cause food poisoning (red circles), owing to the presence of various decay-inducing microorganisms.

A sensory evaluation revealed that the milk at 10 h was spoiled and no longer consumable, according to the smell, taste, and color of the milk. The FL sensors exhibited a visual response to rancid milk, and the FL color changed almost instantly (<5 s). The paper-based method was more sensitive than the film-based method, with better performance than the sensory evaluation, owing to the earlier detection of spoiled milk. The sensing performance may depend on the FL brightness of the N-CQDs and the combination effect, involving N-CQDs and H^+^ [34,35]. The N-CQDs, on paper, displayed excellent FL properties and the combination between H+ and the N-CQDs was more effective and complete than that on the starch-gel film. The steps involved in the developed system include the following: (1) adding milk without pretreatment, which takes <1 min; and (2) visually judging the freshness by referring to the standard colorimetric card, with the aid of only the naked eye and UV light, which can be done within 1 min. This work integrated the N-CQDs into flexible substrates and eliminates the requirement of sophisticated instruments, thereby, reducing complexity. The portable FL sensor could be applied wherever milk needs to be monitored. These results demonstrated that this flexible and eye-readable sensing system can accomplish an easy-to-use visual detection method of milk freshness, with high sensitivity and stability.

## 4. Conclusions

In this study, easy-to-use fluorescence sensors, which were based on acidity-responsive N-CQDs, were designed for the visual sensing of milk freshness. The N-CQDs prepared from poly-amino substances exhibited excellent fluorescence properties and were confirmed to have a regular response to rancid milk, due to the effective fluorescence quenching as the milk’s freshness decreased. The fluorescence intensity showed an excellent linearity (R^2^ = 0.996) with rancid milk over the range 11.6–34.2 °T. Moreover, the fluorescence sensors based on the N-CQDs were fabricated with portability and eye-readability in mind, using paper and starch-gel films as substrates. A significant change in the fluorescence brightness (from bright yellow–green, to deep yellow, and, finally, to blue) was observed by the naked eye as decreasing milk freshness. The practical applications of the results of the fluorescence sensors were consistent with the results obtained by standard methods. This assay was completed within 2 min, via the naked eye under UV light and without any milk pretreatment. These results suggest that a visual sensing of milk freshness can be performed, providing tremendous prospects for the sensitive, rapid, and easy-to-use monitoring of food quality and safety.

## Data Availability

The original contributions presented in the study are included in the article and Supplementary Materials; further inquiries can be directed to the corresponding author.

## Supplementary Materials

The following supporting information can be downloaded at: https://www.mdpi.com/article/10.3390/foods11131855/s1, Table S1: The relative contents of elements C, N, and O in CQDs; Figure S1: (A) XPS pattern of C1s; (B) XPS pattern of N1s; (C) XPS pattern of O1s; Figure S2: (A) effect of CQDs concentration; (B) effect of incubation time. The supplementary materials are available free of charge. Additional information (as noted in the text), the materials and apparatus, the imaging analyzer, XPS data of carbon quantum dots (N-CQDs), optimization of milk freshness detection, and the three equations.
