# Supplementary material for: Easy-to-Use Visual Sensing System for Milk Freshness, Sensitized with Acidity-Responsive N-Doped Carbon Quantum Dots
Source: Foods. 2022 Jun 23;11(13):1855. doi: 10.3390/foods11131855 (PMC9265914; doi:10.3390/foods11131855)
Supplement: Supplementary file 1 [file foods-11-01855-s001.zip › foods-1747701-supplementary.pdf]

Supplementary Materials

Easy-to-Use Visual Sensing System for Milk Freshness, Sensitized with Acidity-Responsive N-Doped Carbon Quantum Dots

Xuetao Hu [1], Xinai Zhang [1], Yanxiao Li [2], Jiyong Shi [1,*], Xiaowei Huang [1], Zhihua Li [1], Junjun Zhang [1], Wenting Li [1], Yiwei Xu [3] and Xiaobo Zou [1]

[1] School of Food and Biological Engineering, Jiangsu University, Zhenjiang 212013, China; xuetaojsu@ujs.edu.cn (X.H.); zhangxinai@mail.ujs.edu.cn (X.Z.); huangxiaowei@ujs.edu.cn (X.H.); lizh@ujs.edu.cn (Z.L.); 2111918019@stmail.ujs.edu.cn (J.Z.); 2112018022@stmail.ujs.edu.cn (W.L.); zou_xiaobo@ujs.edu.cn (X.Z.)

[2] Analyzing and Test Center, Jiangsu University, Zhenjiang 212013, China; 1000003438@ujs.edu.cn

[3] School of Food Science and Technology, Henan University of Science and Technology, Zhengzhou 450001, China; xuyiwei@ujs.edu.cn

* Correspondence: shi_jiyong@ujs.edu.cn

1 Experimental section

Chemical and Materials: O-phenylenediamine, formamide, quinine sulfate (QS), sodium hydroxide, citric acid, lactic acid, disodium hydrogen phosphate, starch, polyvinyl alcohol, and glycerol were obtained from Sinopham Chemical Reagent Co., LTD (Shanghai, China). Citric acid and disodium hydrogen phosphate were mixed in specific proportions to prepare pH buffer solutions over a range of 7.2~ 4.5. Whatman filter paper (Grade 2) was obtained from GE Whatman (Buckinghamshire, UK). Fresh cow milk for the experiments was purchased from a local supermarket (Zhenjiang, China).

Apparatus: Transmission electron microscopy (TEM) images were obtained using TEM (JEOL JEM- 2100, Japan). X-ray diffraction (XRD) patterns were obtained using a powder X-ray diffractometer (D8 ADVANCE, Bruker, Germany). Fourier transform infrared (FT-IR) spectra were recorded using FTIR Spectroscopy (Thermo Scientific Nicolet iS50, USA). X-ray photoelectron spectroscopy (XPS) (Thermo Scientific ESCALAB 250, USA) was performed using an instrument equipped with a monochromatic microspot X-ray beam for elemental analysis. Atomic resolution elemental mapping was performed using a scanning electron microscope (SEM) equipped with an energy dispersive X-ray spectroscopy (EDS) (EDAX TEAM, USA). UV-visible (UV-vis) spectra were obtained by a Rayleigh UV-1601 spectrophotometer (Beifen- Ruili Analytical Instrument Company Limited, China). Fluorescence lifetime was measured using a QuantaMaster™ 40 fluorescence lifetime spectrometer (PTI, USA). Fluorescence spectra were recorded using an F-98 spectrofluorometer (Lengguang Technology Company, China). Fluorescent images were acquired using a fluorescence imaging analyzer assembled in our lab.

2 Additional Tables and Figures:

Table S1 The relative contents of elements C, N, and O in CQDs

Name	Start BE	Peak BE	End BE	Height CPS	Area (P) CPS. eV	Atomic %
C1s	281	285.94	296	9502.2	35911.99	19.85
N1s	394	398.99	408	2044.38	5993.39	2.13
O1s	526	532.49	540	193628.1	1980.66	78.02

Notes: BE- binding energy; CPS- counts per second.

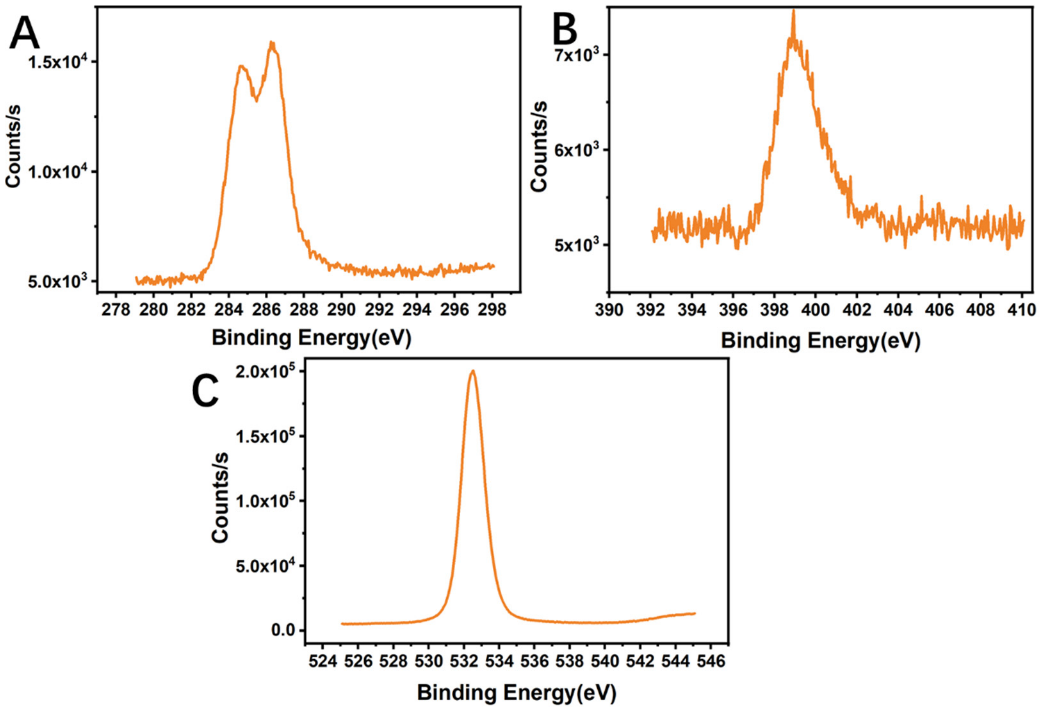

Figure S1 (A) XPS pattern of C1s; (B) XPS pattern of N1s; (C) XPS pattern of O1s

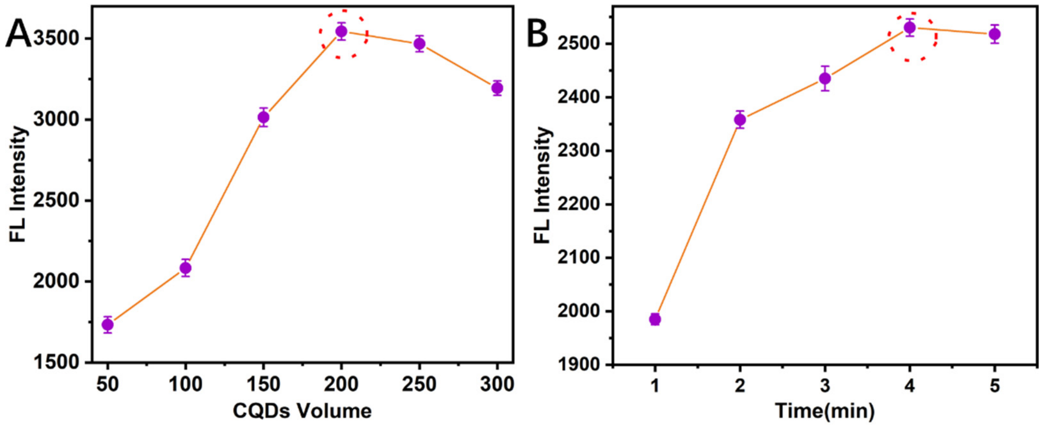

Figure S2 (A) effect of CQDs concentration; (B) effect of incubation time

Reaction parameters (dosage and reaction time) were optimized before establishing the FL method (Fig. S3). CQDs with volumes of 50, 100, 150, 200, 250 and 300 μL were diluted to 2 mL. The concentration of CQDs with the highest fluorescence intensity (arbitrary unit, a. u.) was selected as the optimal concentration. The acid buffer solution (pH=6) was added to the CQDs solution and allowed to stand for 1, 2, 3, 4 or 5 min. The FL intensity reached a maximum at a CQDs dosage of 200 μL diluted to 2 mL (Fig. S3A), and reached a stable state 3 minutes later (Fig. S3B). Therefore, the optimal dosage of CQDs was 200 μL (100 μL/mL), and the optimal reaction time was 3 min.

3 Equations:

$$X(°T) = 100cV/0.1m \quad (1)$$

where X is the acidity of the milk sample (°T), c is the molar concentration of NaOH solution (mol/L), V is the volume (mL) of NaOH solution consumed, m is the quality of the milk (g), 0.1 is the molar concentration (mol/L) of NaOH as defined by the acidity theory.

$$Q_x = Q_{st} (A_{st}/A_x) (I_x/I_{st}) ((\eta^2{}_x)/ (\eta^2{}_{st}) \quad (2)$$

where Q, A and I represent the quantum yield, absorbance and FL intensity, respectively. Q_{st} is equal to 54 %. η is the refractive index of the solvent (1.33 for both solvents, $\eta_{st}/\eta_x = 1$, QS solution is prepared in 0.1 M H_2SO_4). The subscript st and subscript x denote to QS and CQDs, respectively.

$$F_0/F = 1 + K_{SV}[Q] = 1 + k_q\tau_0[Q] \quad (3)$$

where F is the FL intensity of CQDs in the presence of quencher molecules, F_0 is the FL intensity of CQDs in the absence of quencher molecules, Q is the concentration of the quencher, K_{SV} is the SV constant (L/mol), τ_0 is the FL lifetime of CQDs without the quencher, and k_q is the FL quenching rate, which reflects the effect of the quencher on the FL lifetime decay rate constant, generally $k_q = K_{SV}/\tau_0$.
